# Does health-related quality of life change across pregnancy trimesters? A systematic review and meta-analysis

**DOI:** 10.4069/whn.2025.12.02.1

**Published:** 2025-12-31

**Authors:** Hyunjeong Shin, Inhae Cho, Sung-Jin Kim

**Affiliations:** 1 Department of Nursing, Korea University, Seoul, Korea; 2 Department of Nursing, Inje University, Busan, Korea

**Keywords:** Maternal health, Meta-analysis, Pregnancy trimesters, Pregnant people, Quality of life

## Abstract

**Purpose:**

This study aimed to systematically review and synthesize existing evidence on health-related quality of life (HRQOL) across pregnancy trimesters, identifying consistent temporal patterns and domain-specific trajectories in maternal well-being.

**Methods:**

Five databases (PubMed, EMBASE, CINAHL Complete, APA PsycArticles, and RISS) were searched to retrieve English and Korean articles published between January 2001 and May 2023. Two reviewers independently screened and appraised studies using the Joanna Briggs Institute critical appraisal tools. A meta-analysis was performed using STATA 17.0 (StataCorp LLC.), with pairwise comparisons of standardized mean differences used to evaluate trimester-specific variations. Subgroup analyses were performed based on measurement instruments and HRQOL domains (mental vs. physical health).

**Results:**

A total of 13 studies involving 4,809 pregnant participants were included. Overall HRQOL showed no significant differences between the first and second trimesters or between the first and third trimesters. However, HRQOL scores in the third trimester were significantly lower than those in the second trimester (Hedges’ g=–0.42; 95% confidence interval, –0.77 to –0.06). Subgroup analyses revealed that the choice of measurement instruments significantly influenced the detection of changes in HRQOL. Mental health scores improved in mid-pregnancy and remained relatively stable, whereas physical health scores declined after mid-pregnancy, reaching their lowest point in the third trimester.

**Conclusion:**

HRQOL during pregnancy follows distinct temporal patterns, emphasizing the need for trimester-specific maternal care strategies. Early pregnancy may require greater emphasis on mental health support, while late pregnancy may benefit from interventions targeting physical discomfort and functional limitations.

## Introduction

Pregnancy represents a complex and transitional phase in a woman’s life, characterized by a wide range of physiological, psychological, and social changes. These multidimensional shifts can significantly influence women’s health-related quality of life (HRQOL), which serves as a comprehensive indicator of women’s well-being throughout gestation [1-3]. The concept of HRQOL encompasses multiple dimensions including physical functioning, emotional well-being, social relationships, and role limitations, and reflects the subjective experience of health in daily life [1,3-5]. As such, it not only captures pregnant women’s health status but also informs clinical decision-making, predicts healthcare utilization, and contributes to both maternal and neonatal outcomes [4-6].

Several studies have investigated women’s HRQOL during pregnancy and reported changes across gestation [3,7-10]. However, discrepancies remain in the literature regarding how HRQOL changes over time. Wu et al. [3] conducted a large-scale survey and found that HRQOL was highest in the early second trimester, followed by a gradual decline into the third trimester. The authors attributed this pattern to reduced discomfort and improved emotional well-being during mid-pregnancy, compared to the nausea and fatigue of the first trimester and the physical strain of the third [3]. Vachkova et al. [9] also reported that HRQOL peaked in the second trimester and was lowest in the third. Several studies have further observed that women in the third trimester experience the most significant role limitations due to physical discomfort [3,8,9].

Although some studies report fluctuations in HRQOL across trimesters, other recent findings point to a steady downward trend throughout gestation [7,10,11]. A Korean study analyzing data from 4,537 pregnant women found that HRQOL was highest in the first trimester, although the difference compared to the second was slight [11]. A longitudinal study conducted in France observed a gradual reduction in HRQOL from the first trimester to birth, with the most pronounced deterioration occurring in the third trimester [7]. It reported significant declines in physical functioning, vitality, and bodily pain domains, suggesting that increasing physical discomfort and fatigue contribute to diminished well-being over time [7]. Similarly, a cross-sectional pilot study in Morocco found that HRQOL scores were highest in the first trimester and progressively declined through the second and third trimesters [10]. Women in the third trimester reported the greatest limitations in mobility, self-care, and daily activities, along with elevated levels of pain and emotional distress [10].

In contrast to studies reporting significant changes in HRQOL across pregnancy trimesters, some research has found no statistically meaningful differences over time [12]. A study conducted in Jordan revealed that HRQOL scores did not differ significantly between the first, second, and third trimesters, suggesting a relatively stable perception of well-being throughout pregnancy [12].

Taken together, the existing literature presents inconsistent findings regarding the trajectory of HRQOL across gestation. Some studies report increases in emotional distress as pregnancy progresses, while others found fluctuations in emotional well-being. Similarly, physical health outcomes vary widely, with some evidence pointing to gradual deterioration due to increasing physical burden, while other reports suggest stable or fluctuating patterns. These discrepancies may stem from differences in study design, population characteristics, and the measurement instruments used [10,12,13].

Understanding how HRQOL evolves throughout pregnancy is essential for designing responsive trimester-specific maternal care. Despite growing interest in pregnant women’s HRQOL, few studies have systematically synthesized available evidence to clarify its temporal patterns across pregnancy. To address this gap, the present study aims to conduct a systematic review and meta-analysis to comprehensively evaluate HRQOL trajectories across pregnancy trimesters and identify patterns in maternal well-being.

## Methods

**Ethics statement:** This review did not require institutional review board approval as it involved analysis of previously published literature.

This systematic review and meta-analysis aimed to identify changes in HRQOL levels across trimesters of pregnancy. The study was conducted based on the PRISMA (Preferred Reporting Items for Systematic Reviews and Meta-Analyses) 2020 guidelines [14]. The protocol for this systematic review has been registered in the Open Science Framework (Registration DOI: 10.17605/OSF.IO/GX2S4).

### Search strategy

Before systematically searching the literature, we confirmed the MeSH terms, Emtree terms, and free-text terms from the relevant literature based on our research question: “How does women’s HRQOL change across trimesters during pregnancy?” We systematically searched five electronic databases to retrieve relevant articles: PubMed, Embase, CINAHL Complete, APA PsycArticles for English articles, and RISS (Korean research information database) for Korean articles. All observational studies (e.g., cross-sectional, cohort, case-control) that were published from January 2001 to March 2023 were considered. Key search terms included “pregnancy,” “pregnant women,” “quality of life,” “health-related quality of life,” and “HRQOL.” The complete search strategies are presented in Supplementary Figure 1.

### Eligibility criteria

We established the eligibility criteria for identifying articles consistent with the aim of the review, as follows: (1) studies on pregnant women with no complications, (2) studies that evaluated HRQOL across at least two different trimesters, (3) studies that reported available mean HRQOL scores, and (4) were published in peer-reviewed journals. The exclusion criteria were as follows: (1) gray literature such as a conference abstract, (2) articles not published in English or Korean, and (3) ineligible study design (e.g., experimental studies). The research team independently screened and cross-checked all articles.

### Data extraction and synthesis

**Figure 1.**
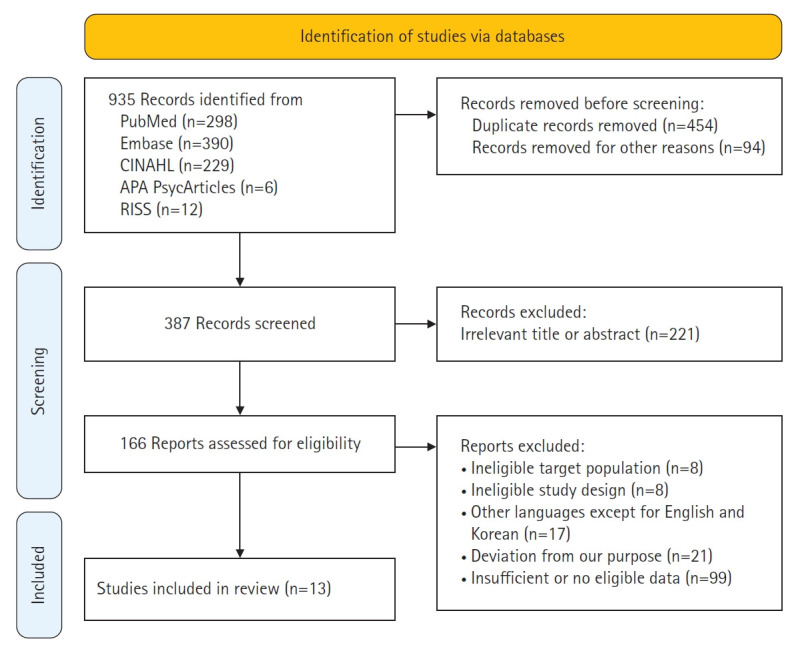
PRISMA (Preferred Reporting Items for Systematic Reviews and Meta-Analyses) 2020 flow diagram of study selection.

Data was extracted by two reviewers (IC and SJK) independently. Duplicates were first deleted using management software (EndNote ver. X9.1; Clarivate, Philadelphia, PA, USA). After duplicate removal, titles and abstracts were reviewed based on predefined criteria, and irrelevant studies were excluded from the review. Ultimately, 13 studies were selected following full-text review, as shown in Figure 1. In cases of disagreement during the selection process, discussions were held to reach consensus; when consensus could not be reached, the third reviewer (HS) made the final decision.

To synthesize the extracted data, we coded the included studies using a predesigned template that captured the following information: study authors, year of publication, country of data collection, study design, study population, participants’ age, sample size, and HRQOL measures. The key results focused on the changes in HRQOL across pregnancy trimesters. We further compare the HRQOL scores between the first and second trimesters, the first and third trimesters, and the second and third trimesters, identifying differences across each stage.

### Study quality assessment

We utilized the Joanna Briggs Institute (JBI) critical appraisal tools for the quality assessment of eligible articles [15]. The JBI’s critical appraisal tools were designed to assist in evaluating the trustworthiness, relevance, and findings of published studies [16]. These tools were applied according to the study type (e.g., cross-sectional, case-control, and cohort designs). Each quality criterion, depending on the study design, consisted of items evaluated as “Yes,” “No,” “Unclear,” or “Not applicable.” The quality of the articles was appraised independently by two reviewers (IC and SJK), and any differences in evaluation were continuously discussed until a consensus was reached. If discrepancies persisted, they were resolved in consultation with a third reviewer (HS).

### Statistical analysis

Data extracted from the included studies were analyzed using STATA ver. 17.0 (StataCorp LLC., College Station, TX, USA). A random-effects model using the DerSimonian–Laird method was applied in meta-analysis. The pooled effect was calculated using inverse-variance weights, in which each study’s weight was defined as the inverse of its within-study variance plus the estimated between-study variance (τ²). This approach accounts for both within-study sampling error and between-study heterogeneity [17]. Standardized mean differences with 95% confidence intervals (CI) were calculated using Hedges’ g to compare HRQOL scores across trimesters. Heterogeneity across studies was assessed using the I^2^ statistic (<40% unimportant heterogeneity, ≥50% substantial heterogeneity) [18]. Sensitivity analysis was conducted to examine the robustness of the findings in this analysis. Subgroup analyses were conducted based on the measurement instruments of HRQOL and their domains.

## Results

### Search results

A total of 935 records were initially identified through searches across the five electronic databases. After removing 454 duplicate entries and excluding 94 records for other reasons (e.g., nonhuman studies, irrelevant topics), 387 articles remained for title and abstract screening. Of these, 221 were excluded based on predefined criteria, resulting in 166 full-text articles assessed for eligibility. Ultimately, 13 studies met the inclusion criteria and were included in the final review (Figure 1).

### Characteristics of the included studies

This review incorporated 13 studies [3,19-30] comprising a total of 4,809 participants. Table 1 presents a summary of the characteristics of these studies. The studies were conducted between 2008 and 2022 across ten countries: China [29,30], Germany [22], Iran [26,27,29], Lebanon [23], Mexico [19], Nigeria [25], Portugal [20], South Korea [28], Taiwan [21], and the United States [24]. Of the included studies, six were cross-sectional [3,23,25-27,29], six were longitudinal [19-22,24,28], and one employed a case-control design [29]. The most studied population consisted of pregnant women aged over 18 years, including subgroups such as singleton pregnancies, nulliparous women undergoing assisted reproductive technology, and individuals with obesity. Sample sizes ranged from 56 to 873 participants, with mean ages ranging from 26.2 to 35.8 years.

HRQOL was most frequently assessed using the Short Form Health Surveys (SF), specifically SF-12 [19,21,25,27] and SF-36 [20,28,30], followed by the World Health Organization Quality of Life-Brief (WHOQOL-BREF) [23,26,29]. Additionally, the Linear Analogue Self-Assessment (LASA) [24], the Quality of Life in Reflux and Dyspepsia questionnaire (QOLRAD) [22], and the EuroQol 5-Dimension 5-Level questionnaire (EQ-5D-5L) [3] each were used once across the included studies.

Methodological quality was evaluated using the JBI critical appraisal tools, with scores ranging from 63.64 to 100, indicating a moderate to high level of quality across the included studies (Table 1).

### Longitudinal patterns of health-related quality of life during pregnancy

**Figure 2.**
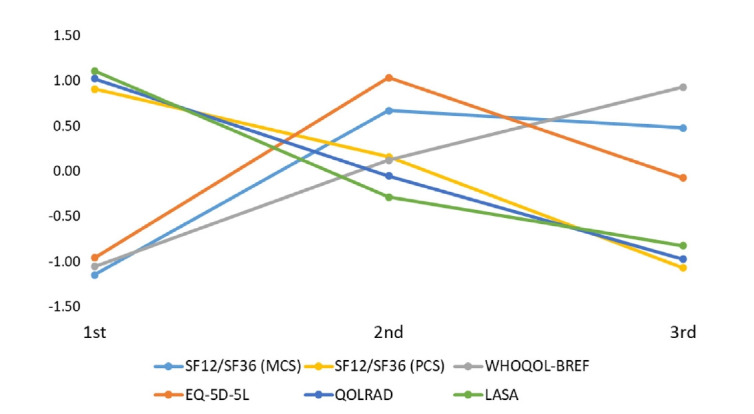
Trajectories of health-related quality of life across pregnancy trimesters by measurement instrument. EQ-5D-5L: EuroQol 5-Dimension 5-Level questionnaire; LASA: Linear Analogue Self-Assessment; MCS: mental component summary; PCS: physical component summary; QOLRAD: Quality of Life in Reflux and Dyspepsia; SF-12: 12-Item Short Form Health Survey; SF-36: 36-Item Short Form Health Survey; WHOQOL-BREF: World Health Organization Quality of Life-Brief.

Longitudinal patterns of overall HRQOL during pregnancy were depicted across trimesters for each measurement instrument (Figure 2). For each trimester, overall HRQOL scores were calculated as sample size-weighted pooled means. To facilitate comparison across different instruments, weighted means were standardized using z-scores.

The results showed that HRQOL trajectories varied by measurement instrument. The mental component summary (MCS) scores of the SF-12 and SF-36, as well as the EQ-5D-5L scores, peaked in the second trimester and declined in the third trimester. The WHOQOL-BREF showed gradual improvement across trimesters, whereas the physical component summary (PCS) scores of the SF-12 and SF-36, as well as the QOLRAD and LASA scores, demonstrated a steady decline.

### Pairwise comparisons of health-related quality of life across trimesters

**Figure 3.**
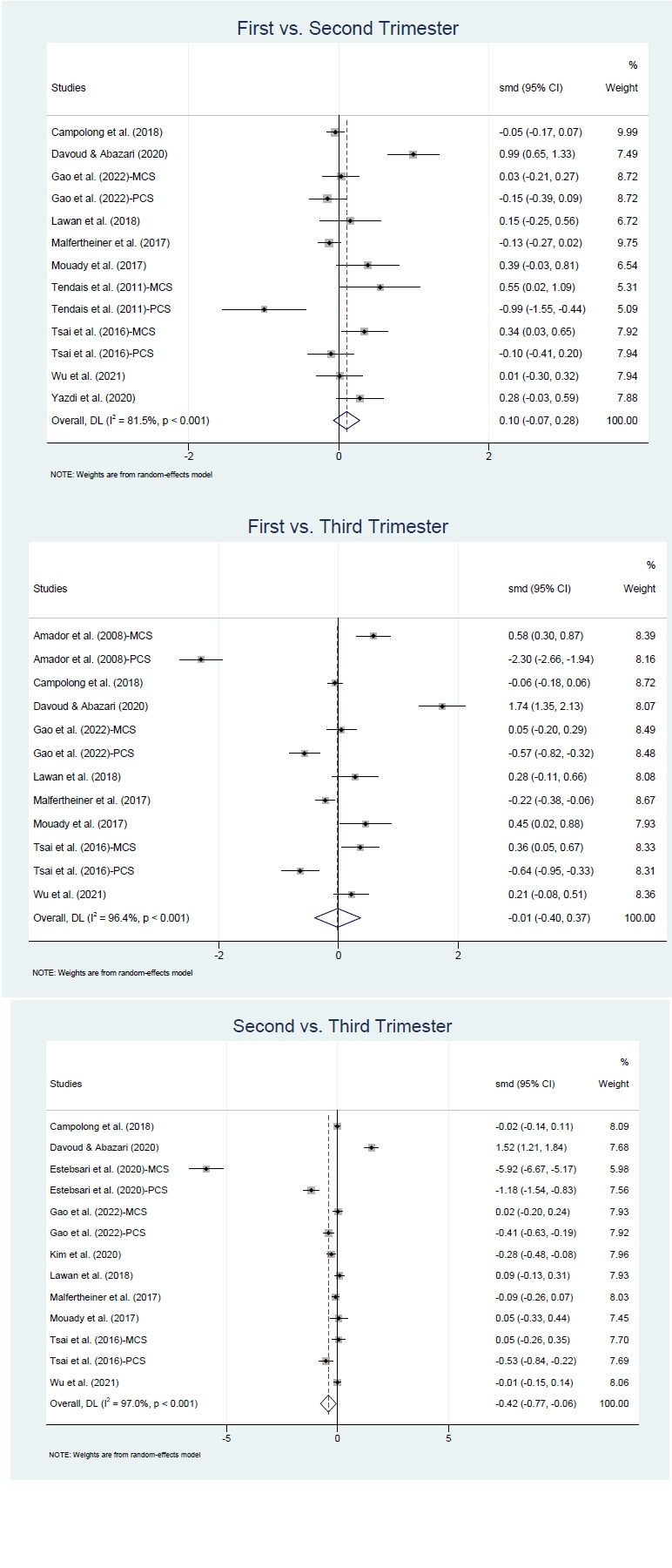
Forest plots of health-related quality of life across trimesters.

HRQOL scores were compared across trimesters using data from the included studies (Figure 3). To identify trimester-specific differences, pairwise comparisons were conducted using standardized mean differences: first versus second trimester, first versus third trimester, and second versus third trimester. Ten studies contributed data for the first versus second trimester comparison [3,20-26,29,30], nine for the first versus third trimester [3,19,21-26,30], and ten for the second versus third trimester [3,21-28,30].

No statistically significant differences in HRQOL scores were observed between the first and second trimesters (Hedges’ g=0.10; 95% CI, –0.07 to 0.28; I^2^=81.5%, *p*<.001), nor between the first and third trimesters (Hedges’ g=–0.01; 95% CI, –0.40 to 0.37; I^2^=96.4%, *p*<.001). In contrast, scores in the third trimester were significantly lower than those in the second trimester (Hedges’ g=–0.42; 95% CI, –0.77 to –0.06; I^2^=97.0%, *p*<.001). High heterogeneity was observed across all comparisons.

### Subgroup analyses of health-related quality of life by measurement instruments and domains

**Figure 4.**
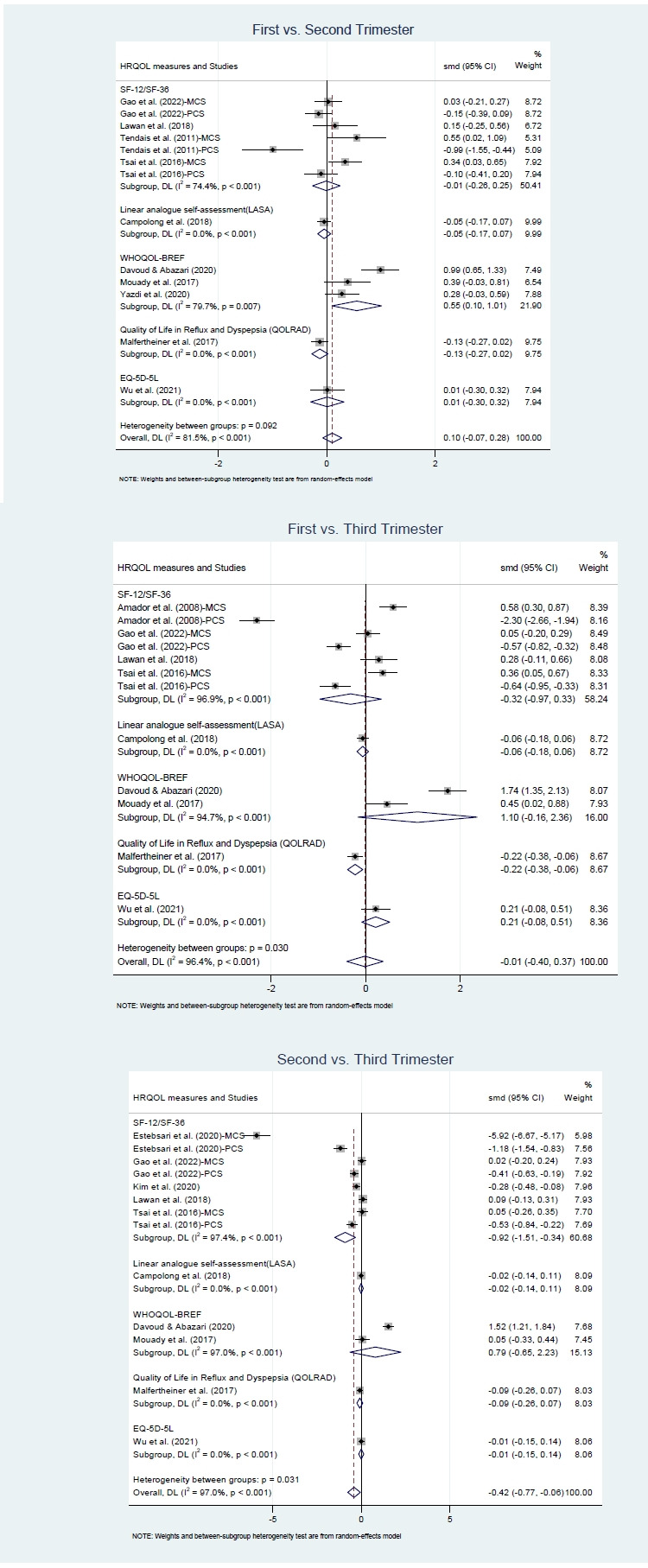
Forest plots of subgroup analyses: measurement across trimesters.

Subgroup analyses were performed to examine variations in HRQOL across both measurement instruments and domains. In the instrument-specific analysis, the WHOQOL-BREF scores showed significant differences between the first and second trimesters (Hedges’ g=0.55; 95% CI, 0.10–1.01; I^2^=79.7%, *p*<.001). The SF-12 and SF-36 demonstrated a significantly larger effect size in the comparison between the second and third trimesters (Hedges’ g=–0.92; 95% CI, –1.51 to –0.34; I^2^=97.4%, *p*<.001), whereas other instruments (including LASA, QOLRAD, and EQ-5D-5L) yielded non-significant effect sizes (Figure 4).

**Figure 5.**
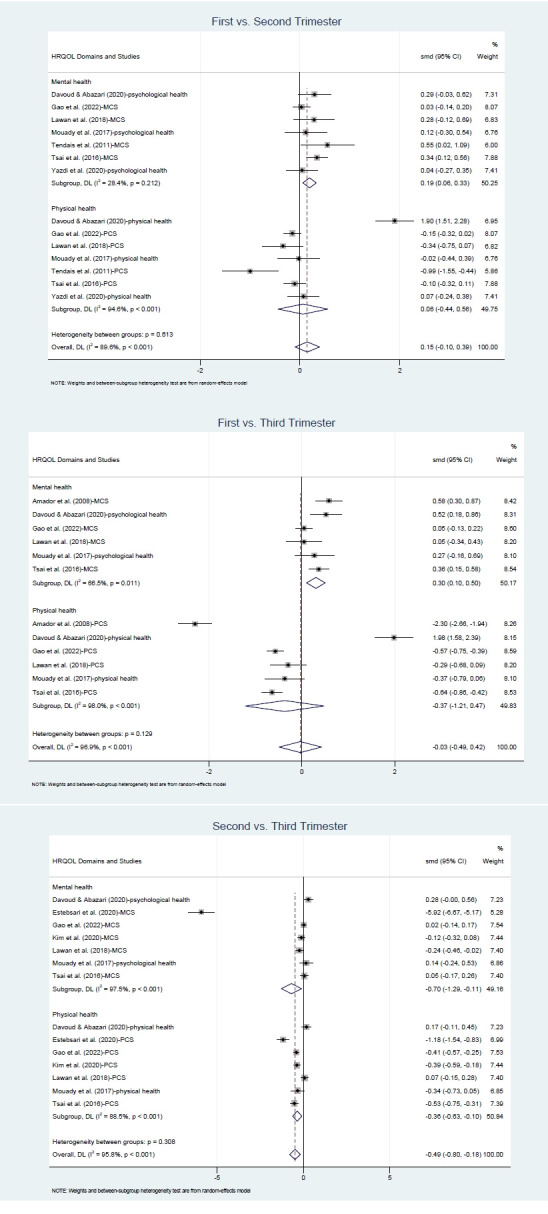
Forest plots of subgroup analyses: health-related quality of life (HRQOL) domains across trimesters.

For the 10 studies that assessed both physical and mental health domains of HRQOL using the SF-12, SF-36, or WHO QOLBREF, an additional subgroup analysis was performed to explore domain-specific variations, focusing on the MCS/psychological domain and PCS/physical domain. For the mental/psychological domain, scores significantly increased from the first to the second trimester (Hedges’ g=0.19; 95% CI, 0.06–0.33; I^2^=28.4%, *p*=.212), but a significant decrease was observed between the second and the third trimesters (Hedges’ g=–0.70; 95% CI, –1.29 to –0.11; I^2^=97.5%, *p*<.001). Psychological scores in the third trimester were significantly higher than those in the first trimester (Hedges’ g=0.30; 95% CI, 0.10–0.50; I^2^=66.5%, *p*=.011). For the physical health domain, however, a significant decline was observed only in the comparison between the second and third trimesters (Hedges’ g=–0.36; 95% CI, –0.63 to –0.10; I^2^=88.5%, *p*<.001). Comparisons between the first and second trimesters, as well as between the first and the third trimesters, were not statistically significant (Figure 5).

In addition, meta-regression was conducted to explore potential sources of between-study heterogeneity. Study design (cross-sectional, longitudinal, and case control) was examined as a moderator; however, it did not significantly explain the observed heterogeneity (Supplementary Table 1). This suggests that differences in study design alone were insufficient to explain the high degree of heterogeneity observed.

### Assessment of publication bias and robustness of findings

To assess potential publication bias, a funnel plot was generated. The plot exhibited an overall symmetric distribution with mild right-sided dispersion and one extreme outlier (Supplementary Figure 2). Begg’s rank correlation test revealed no statistically significant evidence of small-study effects (z=–1.77, *p*=.0995), indicating that publication bias was unlikely among the included studies.

Sensitivity analyses were performed to evaluate the robustness of the findings. A leave-one-out approach was applied, whereby each study was sequentially excluded to assess its influence on the overall effect size. The pooled mean differences remained consistent with the original estimates, and most comparisons retained statistical significance following the sensitivity analysis (Supplementary Figure 3). These results suggest that the findings are stable and not unduly influenced by any single study.

## Discussion

This systematic review and meta-analysis synthesized findings from 13 studies encompassing 4,809 participants to examine trimester-specific variations in HRQOL during pregnancy. Through pooled mean trajectories and pairwise comparisons of standardized mean differences, we identified specific patterns in HRQOL across trimesters.

Our pooled trajectories revealed that the pattern differed depending on the measurement instrument. In contrast to a prospective cohort study that reported the lowest quality of life between the 4th and 8th months of pregnancy [7], our findings showed that HRQOL trajectories varied across instruments. While MCS and EQ-5D-5L demonstrated higher HRQOL levels in the second trimester, others showed gradual improvement or consistent decline. This suggests that HRQOL does not follow a consistent pattern across pregnancy.

The comparative analysis of overall HRQOL across trimesters revealed no statistically significant differences between the first and second trimesters as well as between the first and third trimesters. However, scores in the third trimester were significantly lower than those in the second, consistent with previous literature that describes a mid-pregnancy improvement followed by a late-pregnancy decline [3,5,8]. Several factors may account for this pattern. The third trimester is often marked by significant limitations in social functioning and daily activities [1]. Previous review studies have indicated that anxiety rates increased from 18.2% in the first trimester to 24.6% in the third trimester [31], which may impair pregnant women’s sleep quality and overall well-being [3,21]. Taken together, the convergence of physical discomfort, heightened anxiety, sleep disturbances, and reduced social and role functioning provides a coherent explanation for the observed decline in HRQOL during late pregnancy.

Given the substantial heterogeneity observed across studies, this research conducted subgroup analyses from two perspectives: measurement instruments and HRQOL domains. Subgroup analyses revealed that the choice of instruments significantly influenced the detection of HRQOL changes. In the present study, as seven of the 13 studies utilized the Short Form Health Survey to assess HRQOL, the trajectory of overall HRQOL across trimesters observed is primarily based on these studies. However, the findings showed instrument-dependent variation in pairwise comparisons. For example, WHOQOL-BREF detected improvements from the first to the second trimester, whereas SF-12/SF-36 identified declines from the second to the third trimester. In contrast, other tools showed no differences. These inconsistencies across instruments raise a critical question regarding which tools most accurately capture the lived experiences and HRQOL of pregnant women. The divergent trajectories observed suggest that although generic measures are widely validated and commonly used in maternal health research, they may not be sufficiently sensitive to pregnancy-specific concerns, thereby complicating the interpretation of results and limiting comparability across studies.

Another subgroup analysis by HRQOL domain revealed divergent trajectories for mental and physical health. For the mental health domain, second-trimester scores tended to be higher than those in the first and third trimesters. These results are consistent with previous findings showing that early psychological adaptation challenges [32,33] and late-pregnancy stressors [34] can negatively affect maternal well-being. Similarly, Amiel Castro et al. [35] reported that depressive symptoms were more prevalent in the first and third trimesters than in the second. In contrast, physical health scores in our subgroup analysis showed a statistically significant decrease only between the second and third trimesters. Physical symptoms and functional burden may increase after mid-pregnancy, leading to progressive restrictions in daily functioning [1], with the greatest impact typically observed during the third trimester [36]. These findings suggest that psychological and physical aspects of maternal well-being follow distinct temporal patterns during pregnancy, indicating the need for trimester-specific care strategies. Early pregnancy appears to require particular attention to mental health support. Psychological difficulties during this period may lead to poorer maternal–fetal attachment [37,38] and may influence women’s preferences regarding mode of delivery [39]. Routine screening for depression and anxiety with subsequent referral to counseling or cognitive-behavioral therapy has been shown to improve maternal outcomes [40]. Structured prenatal education programs incorporating stress management techniques, such as mindfulness [41] and relaxation training [42], further enhance psychological well-being. Integrating mental health support into routine antenatal care may help women receive timely assistance. In contrast, late pregnancy may benefit from interventions that reduce physical discomfort and functional limitations. Findings from prior reviews suggest that exercise interventions are particularly effective in enhancing quality of life during the later stages of pregnancy, especially the second and third trimesters [43]. Prenatal exercise programs may reduce musculoskeletal pain and improve sleep quality [44]. Promoting regular exercise may therefore help women stay more comfortable and active as pregnancy progresses.

This meta-analysis has several limitations that warrant consideration. First, substantial heterogeneity was observed across included studies. Such high heterogeneity is frequently reported in HRQOL meta-analyses [45,46] and is often attributed to the inherently multidimensional nature of HRQOL. Although subgroup analyses and meta-regression were conducted to explore sources of variation in our study, residual heterogeneity may still affect the generalizability of the findings. Second, the reliance on generic HRQOL instruments in many studies may have limited the sensitivity to detect pregnancy-specific concerns, such as concerns about the fetus, body image changes, and childbirth-related anxiety. Third, the trimester-based categorization assumes uniformity within each period, potentially overlooking more granular fluctuations in women’s HRQOL. Fourth, although Begg’s test was not significant and trim-and-fill analysis imputed no missing studies, the possibility of publication bias cannot be completely excluded. Lastly, cultural and healthcare system differences across study settings were not systematically addressed, which may influence both HRQOL perceptions and access to care.

To address the limitations identified in this meta-analysis, future research should prioritize the use of pregnancy-specific HRQOL instruments to ensure conceptual relevance and improve sensitivity to gestational changes. Longitudinal designs with more granular time frames, such as monthly assessments, may better capture dynamic fluctuations in women’s HRQOL that trimester-based categorizations overlook. Additionally, cross-cultural studies are needed to explore how sociocultural contexts and healthcare systems influence perceptions of HRQOL. Finally, integrating HRQOL data with clinical outcomes could facilitate the development of more responsive and individualized maternal care strategies.

In conclusion, this study identified trimester-specific variations in maternal HRQOL, with mid-pregnancy emerging as a relatively favorable period and late pregnancy marked by significant declines. These findings indicate the importance of tailoring antenatal care to the distinct psychological needs of women in early pregnancy and to the physical challenges that intensify in late pregnancy. Addressing mental health concerns in the first trimester and mitigating physical discomfort in the third trimester may help optimize maternal well-being.

## Figures and Tables

**Table 1.** Characteristics of the 13 included studies

Authors [Ref] (year)	Country	Study type	Number	Study populations	Age (yr), mean±SD	HRQOL measures	Possible score range	Quality score
Amador et al. [19] (2008)	Mexico	Longitudinal study	220	Pregnant women	Obese women: 28.4±5.0	SF-12	PCS: 0–100	100.00
					Non-obese women: 26.2±4.0		MCS: 0–100	
Tendais et al. [20] (2011)	Portugal	Longitudinal study	56	Pregnant women at 10–15 weeks	30.4±4.0	SF-36	PCS: 0–100	75.00
							MCS: 0–100	
Tsai et al. [21] (2016)	Taiwan	Longitudinal study	164	Pregnant women in the first trimester aged ≥18 years	32.7±4.0	SF-12	PCS: 0–100	100.00
							MCS: 0–100	
Fill Malfertheiner et al. [22] (2017)	Germany	Longitudinal study	510	Pregnant women in the first trimester	28.3±5.1	QOLRAD	1–7	63.64
Mourady et al. [23] (2017)	Lebanon	Cross-sectional study	141	Pregnant women aged ≥18 years	30.5±5.2	WHOQOL-BREF	4–20 per domain	87.50
Campolong et al. [24] (2018)	USA	Longitudinal study	572	Singleton pregnant women aged 18–45 years	30.5±4.5	LASA	0–10	72.73
Lawan et al. [25] (2018)	Nigeria	Cross-sectional study	398	Pregnant women	27.9±5.2	SF-12	PCS: 0–100	87.50
							MCS: 0–100	
Davoud and Abazari [26] (2020)	Iran	Cross-sectional study	256	Pregnant women aged ≥18 years	30.2±5.7	WHOQOL-BREF	4–20 per domain	87.50
Estebsari et al. [27] (2020)	Iran	Cross-sectional study	300	Pregnant women in the second and third trimesters	32.9±6.1	SF-12	PCS: 0–100	75.00
							MCS: 0–100	
Kim et al. [28] (2020)	South Korea	Longitudinal study	385	Pregnant women in the second and third trimesters	The second trimester: 32.9±3.9	SF-36	PCS: 0–100	62.50
					The third trimester: 33.0±3.9		MCS: 0–100	
Sarafraz Yazdi et al. [29] (2020)	Iran	Case-control study	80	Nulliparous women conceived with ART and nulliparous women conceived spontaneously	ART: 29.4±4.4	WHOQOL-BREF	4–20 per domain	80.00
					Spontaneous pregnancy: 29.6±5.0			
Wu et al. [3] (2021)	China	Cross-sectional study	873	Singleton pregnant women	35.8±4.9	EQ-5D-5L	–0.066 to 1	87.50
							(utility value)	
Gao et al. [30] (2022)	China	Cross-sectional study	854	Singleton pregnant women aged ≥18 years	33.0±5.3	SF-36	PCS: 0–100	100.00
							MCS: 0–100	

ART: Assisted reproductive technology; EQ-5D-5L: EuroQol 5-Dimension 5-Level questionnaire; HRQOL: health-related quality of life; LASA: Linear Analogue Self-Assessment; MCS: mental component summary; PCS: physical component summary; QOLRAD: Quality of Life in Reflux and Dyspepsia; SF-12: 12-Item Short Form Health Survey; SF-36: 36-Item Short Form Health Survey; WHOQOL-BREF: World Health Organization Quality of Life-Brief.

## Supplementary materials

Further details on supplementary materials are presented online (available at https://doi.org/10.4069/whn.2025.12.02.1).

Supplementary Table 1. Meta-regression examining the moderating effect of study design

Supplementary Figure 1. Electronic search strategy in electronic databases.

Supplementary Figure 2. Funnel plot of the included studies.

Supplementary Figure 3. Leave-one-out analysis: sensitivity analysis.

## References

[1] Lagadec N, Steinecker M, Kapassi A, Magnier AM, Chastang J, Robert S (2018). Factors influencing the quality of life of pregnant women: a systematic review. BMC Pregnancy Childbirth.

[2] Vahedi S (2010). World Health Organization Quality-of-Life Scale (WHOQOL-BREF): analyses of their item response theory properties based on the graded responses model. Iran J Psychiatry.

[3] Wu H, Sun W, Chen H, Wu Y, Ding W, Liang S (2021). Health-related quality of life in different trimesters during pregnancy. Health Qual Life Outcomes.

[4] Bai G, Korfage IJ, Mautner E, Raat H (2019). Associations between maternal health-related quality of life during pregnancy and birth outcomes: the Generation R study. Int J Environ Res Public Health.

[5] Boutib A, Chergaoui S, Marfak A, Hilali A, Youlyouz-Marfak I (2022). Quality of life during pregnancy from 2011 to 2021: systematic review. Int J Womens Health.

[6] Yamamoto-Hanada K, Pak K, Saito-Abe M, Sato M, Ohya Y (2021). Better maternal quality of life in pregnancy yields better offspring respiratory outcomes: a birth cohort. Ann Allergy Asthma Immunol.

[7] Morin M, Claris O, Dussart C, Frelat A, de Place A, Molinier L (2019). Health-related quality of life during pregnancy: a repeated measures study of changes from the first trimester to birth. Acta Obstet Gynecol Scand.

[8] Kazemi A, Dadkhah A, Torabi F (2022). Changes of health related quality of life during pregnancy based on pregnancy context: a prospective study. Arch Public Health.

[9] Vachkova E, Jezek S, Mares J, Moravcova M (2013). The evaluation of the psychometric properties of a specific quality of life questionnaire for physiological pregnancy. Health Qual Life Outcomes.

[10] Boutib A, Chergaoui S, Azizi A, Saad EM, Hilali A, Youlyouz Marfak I (2023). Health-related quality of life during three trimesters of pregnancy in Morocco: cross-sectional pilot study. EClinicalMedicine.

[11] Lee J, Han YJ, Ryu HM, Kwak DW, Kim MY, Cha DH (2019). A study on the relationship between the health behavior and health-related quality of life during pregnancy and postpartum. J Heal Technol Assess.

[12] Alzboon G, Vural G (2019). Factors influencing the quality of life of healthy pregnant women in North Jordan. Medicina (Kaunas).

[13] Brekke M, Berg RC, Amro A, Glavin K, Haugland T (2022). Quality of life instruments and their psychometric properties for use in parents during pregnancy and the postpartum period: a systematic scoping review. Health Qual Life Outcomes.

[14] Page MJ, McKenzie JE, Bossuyt PM, Boutron I, Hoffmann TC, Mulrow CD (2021). The PRISMA 2020 statement: an updated guideline for reporting systematic reviews. BMJ.

[15] Munn Z, Barker TH, Moola S, Tufanaru C, Stern C, McArthur A (2020). Methodological quality of case series studies: an introduction to the JBI critical appraisal tool. JBI Evid Synth.

[16] Moola S, Munn Z, Tufanaru C, Aromataris E, Sears K, Sfetcu R, Aromataris E, Munn Z (2020). JBI manual for evidence synthesis.

[17] DerSimonian R, Laird N (2015). Meta-analysis in clinical trials revisited. Contemp Clin Trials.

[18] Wang Y, DelRocco N, Lin L (2024). Comparisons of various estimates of the I^2^ statistic for quantifying between-study heterogeneity in meta-analysis. Stat Methods Med Res.

[19] Amador N, Juárez JM, Guízar JM, Linares B (2008). Quality of life in obese pregnant women: a longitudinal study. Am J Obstet Gynecol.

[20] Tendais I, Figueiredo B, Mota J, Conde A (2011). Physical activity, health-related quality of life and depression during pregnancy. Cad Saude Publica.

[21] Tsai SY, Lee PL, Lin JW, Lee CN (2016). Cross-sectional and longitudinal associations between sleep and health-related quality of life in pregnant women: a prospective observational study. Int J Nurs Stud.

[22] Fill Malfertheiner S, Seelbach-Göbel B, Costa SD, Ernst W, Reuschel E, Zeman F (2017). Impact of gastroesophageal reflux disease symptoms on the quality of life in pregnant women: a prospective study. Eur J Gastroenterol Hepatol.

[23] Mourady D, Richa S, Karam R, Papazian T, Hajj Moussa F, El Osta N (2017). Associations between quality of life, physical activity, worry, depression and insomnia: a cross-sectional designed study in healthy pregnant women. PLoS One.

[24] Campolong K, Jenkins S, Clark MM, Borowski K, Nelson N, Moore KM (2018). The association of exercise during pregnancy with trimester-specific and postpartum quality of life and depressive symptoms in a cohort of healthy pregnant women. Arch Womens Ment Health.

[25] Lawan A, Awotidebe AW, Oyeyemi AL, Rufa'i AA, Oyeyemi AY (2018). Relationship between physical activity and health related quality of life among pregnant women. Afr J Reprod Health.

[26] Davoud A, Abazari M (2020). The relationship between quality of life and physical activity, worry, depression, and insomnia in pregnant women. Iran J Psychiatry.

[27] Estebsari F, Kandi ZRK, Bahabadi FJ, Filabadi ZR, Estebsari K, Mostafaei D (2020). Health-related quality of life and related factors among pregnant women. J Educ Health Promot.

[28] Kim KT, Cho YW, Bae JG (2020). Quality of sleep and quality of life measured monthly in pregnant women in South Korea. Sleep Breath.

[29] Sarafraz Yazdi M, Nasiri R, Gharaei Jomei M, Sarafraz Yazdi S (2020). Quality of life and general health in pregnant women conceived with assisted reproductive technology: a case-control study. Int J Fertil Steril.

[30] Gao LL, Yang JP, Wang DN, Sun K (2022). Health related quality of life in Chinese pregnant women at advanced maternal age: a cross-sectional study. J Reprod Infant Psychol.

[31] Dennis CL, Falah-Hassani K, Shiri R (2017). Prevalence of antenatal and postnatal anxiety: systematic review and meta-analysis. Br J Psychiatry.

[32] Huo L, Li X, Yu X, Nisar A, Yang L (2024). Profiles and associated factors of prenatal psychological symptoms and their association with health-related quality of life among pregnant women: a cross-sectional study. BMJ Open.

[33] Huang X, Wang Y, Wang Y, Guo X, Zhang L, Wang W (2023). Prevalence and factors associated with trajectories of antenatal depression: a prospective multi-center cohort study in Chengdu, China. BMC Pregnancy Childbirth.

[34] Zhang H, Zhang Q, Gao T, Kong Y, Qin Z, Hu Y (2019). Relations between stress and quality of life among women in late pregnancy: the parallel mediating role of depressive symptoms and sleep quality. Psychiatry Investig.

[35] Amiel Castro RT, Pinard Anderman C, Glover V, O'Connor TG, Ehlert U, Kammerer M (2017). Associated symptoms of depression: patterns of change during pregnancy. Arch Womens Ment Health.

[36] Chang SR, Chen KH, Lin MI, Lin HH, Huang LH, Lin WA (2014). A repeated measures study of changes in health-related quality of life during pregnancy and the relationship with obstetric factors. J Adv Nurs.

[37] Ko E, Lee Y (2025). Influencing factors and consequences of maternal-fetal attachment among pregnant women in East Asia: a scoping review. J Transcult Nurs.

[38] Rubertsson C, Hellström J, Cross M, Sydsjö G (2014). Anxiety in early pregnancy: prevalence and contributing factors. Arch Womens Ment Health.

[39] Andersson L, Sundström-Poromaa I, Bixo M, Wulff M, Bondestam K, åStröm M (2003). Point prevalence of psychiatric disorders during the second trimester of pregnancy: a population-based study. Am J Obstet Gynecol.

[40] Agampodi T, Amarasinghe G, Wickramasinghe A, Wickramasinghe N, Warnasekara J, Jayasinghe I (2023). Incorporating early pregnancy mental health screening and management into routine maternal care: experience from the Rajarata Pregnancy Cohort (RaPCo), Sri Lanka. BMJ Glob Health.

[41] Hall HG, Beattie J, Lau R, East C, Anne Biro M (2016). Mindfulness and perinatal mental health: a systematic review. Women Birth.

[42] Leutenegger V, Grylka-Baeschlin S, Wieber F, Daly D, Pehlke-Milde J (2022). The effectiveness of skilled breathing and relaxation techniques during antenatal education on maternal and neonatal outcomes: a systematic review. BMC Pregnancy Childbirth.

[43] Liu N, Gou WH, Wang J, Chen DD, Sun WJ, Guo PP (2019). Effects of exercise on pregnant women's quality of life: a systematic review. Eur J Obstet Gynecol Reprod Biol.

[44] Choong SYX, Tan XYJ, Cheng LJ, Lau Y (2022). Effectiveness of exercise in improving sleep outcomes among perinatal women: a systematic review and meta-analysis of randomised controlled trials. Behav Sleep Med.

[45] Phyo AZZ, Freak-Poli R, Craig H, Gasevic D, Stocks NP, Gonzalez-Chica DA (2020). Quality of life and mortality in the general population: a systematic review and meta-analysis. BMC Public Health.

[46] Rowsell A, Sodergren SC, Vassiliou V, Darlington AS, Guren MG, Alkhaffaf B (2022). Systematic review of health-related quality of life (HRQoL) issues associated with gastric cancer: capturing cross-cultural differences. Gastric Cancer.

