# Supplementary material for: Does health-related quality of life change across pregnancy trimesters? A systematic review and meta-analysis
Source: Womens Health Nurs. 2025 Dec 31;31(4):320–34. doi: 10.4069/whn.2025.12.02.1 (PMC12844566; doi:10.4069/whn.2025.12.02.1)
Supplement: Supplementary Table 1. — Meta-regression examining the moderating effect of study design [file whn-2025-12-02-1-Supplementary-Table-1.pdf]

Supplementary Table 1. Meta-regression examining the moderating effect of study design

	Coefficient (β)	SE	95% CI	*p*
The first vs. second trimester				
Case-control study	0.33	0.48	−0.74 to 1.40	0.508
Cross-sectional study	0.27	0.26	−0.30 to 0.85	0.314
The first vs. third Trimester				
Cross-sectional study	0.73	0.52	−0.44 to 1.89	0.195
The first vs. second trimester				
Cross-sectional study	−.53	0.99	−2.73 to 1.67	0.607
