# Supplementary material for: Does health-related quality of life change across pregnancy trimesters? A systematic review and meta-analysis
Source: Womens Health Nurs. 2025 Dec 31;31(4):320–34. doi: 10.4069/whn.2025.12.02.1 (PMC12844566; doi:10.4069/whn.2025.12.02.1)
Supplement: Supplementary Figure 1. — Electronic search strategy in electronic databases. [file whn-2025-12-02-1-Supplementary-Figure-1.pdf]

PubMed
("Pregnancy"[Title] OR "pregnant women"[Title]) AND ("quality of life"[Title] OR "health related quality of life"[Title] OR "HRQOL"[Title])
Results: 298 (23.03.16)

Embase
('pregnant women':ti OR 'pregnancy':ti) AND ('health related quality of life':ti OR 'hrqol':ti OR 'quality of life':ti) AND [2001-2023/py
Results: 390 (23.03.16)

CINAHL complete
(TI 'pregnancy' OR TI 'pregnant women') AND (TI 'health related quality of life' OR TI 'hrqol' OR TI 'quality of life')
Results: 229 (23.03.16)

APA PsycArticles
('pregnancy' OR 'pregnant women') AND ('quality of life' OR 'health-related quality of life' OR 'hrqol')
Results: 6 (23.03.16)

RISS
(건강관련 삶의 질|삶의 질) (임신|임부|임산부)

Results : 12 (23.03.16.)

Supplementary Figure 1. Electronic search strategy in electronic databases.
