# Supplementary figures and images for: Does health-related quality of life change across pregnancy trimesters? A systematic review and meta-analysis
Source: Womens Health Nurs. 2025 Dec 31;31(4):320–34. doi: 10.4069/whn.2025.12.02.1 (PMC12844566; doi:10.4069/whn.2025.12.02.1)

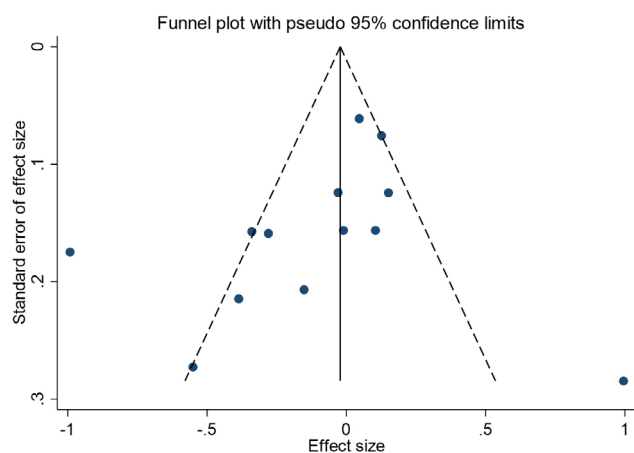

Supplementary Figure 2. Funnel plot of the included studies.

Supplement: Supplementary Figure 2. — Funnel plot of the included studies. [file whn-2025-12-02-1-Supplementary-Figure-2.pdf]

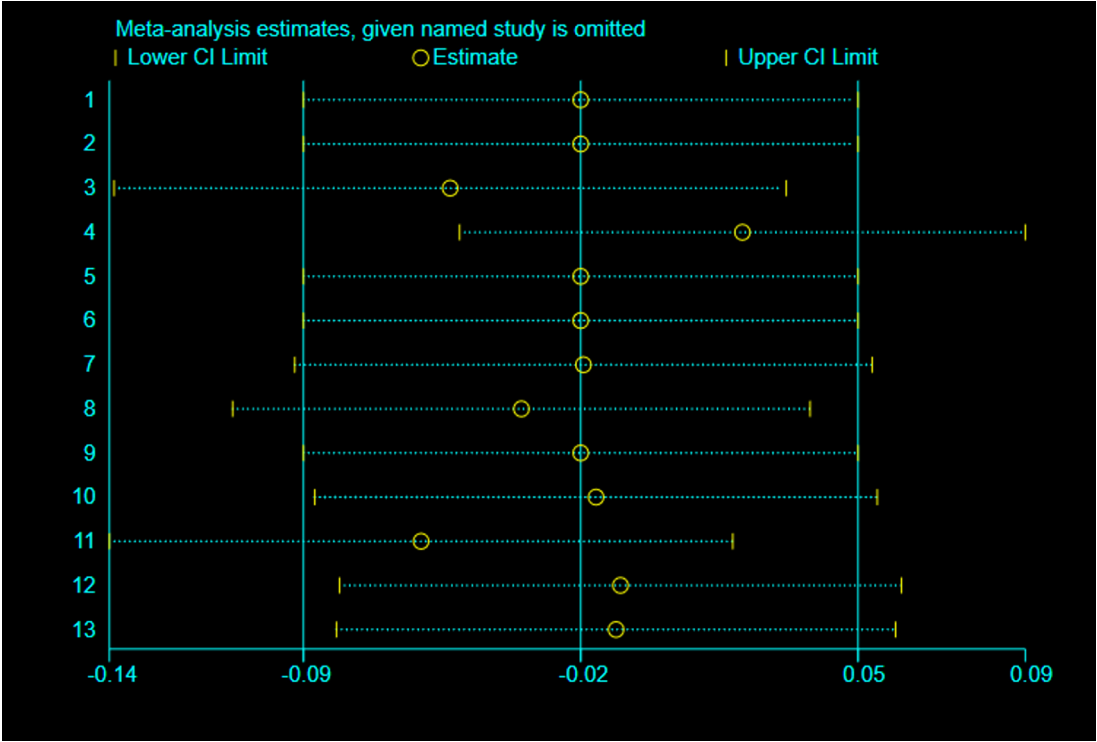

Supplementary Figure 3. Leave-one-out analysis: sensitivity analysis.

Supplement: Supplementary Figure 3. — Leave-one-out analysis: sensitivity analysis. [file whn-2025-12-02-1-Supplementary-Figure-3.pdf]
